# Prevalence of Dental Malocclusions in Different Geographical Areas: Scoping Review

**DOI:** 10.3390/dj9100117

**Published:** 2021-10-11

**Authors:** Niccoló Cenzato, Anna Nobili, Cinzia Maspero

**Affiliations:** Department of Biomedical, Surgical and Dental Sciences, School of Dentistry, University of Milan, UOC Maxillo-Facial Surgery and Dentistry, Fondazione IRCCS Ca Granda, Ospedale Maggiore Policlinico, 20122 Milan, Italy; anna.nobili@gmail.com (A.N.); cinzia.maspero@unimi.it (C.M.)

**Keywords:** prevalence, malocclusion, dental, geographical areas, angle classes

## Abstract

The World Health Organization (WHO) considers malocclusion one of the most important oral health problems, after caries and periodontal disease. Its prevalence is highly variable and is estimated to be between 39% and 93% in children and adolescents. Due to the importance of malocclusions in dentistry, the aim of our review is to assess the frequency of malocclusions among different geographical regions. A literature research was performed through the Pubmed, Medline, Scopus, Web of Science, LILACS, Open Grey and Cochrane Library databases. The “PRISMA” guidelines were used for the following review. Fourteen studies were analysed for this review. Class I was found most frequently, followed by class II and finally class III. Considering the other anomalies, crowding was one of the most frequent with a prevalence of up to 84%, followed by spacing, which reached a frequency of 60%. Prevalence of crossbite and openbite was quite variable, while the evaluation of deepbite revealed more uniform values. The prevalence varied widely for most of the types of malocclusion in relation to the different populations, which suggests a role of genetics and environmental influences, typical of each population in determining dental problems.

## 1. Introduction

The WHO considers malocclusion one of the most important oral health problem, after caries and periodontal disease [1]. Its prevalence is highly variable and is estimated to be between 39% and 93% in children and adolescents [2,3,4]. This prevalence range is very wide and heterogeneous. This inhomogeneity may be due to ethnic and age differences of patients considered in studies, assessing the prevalence of malocclusion [5,6].

Malocclusions can occur in three different spatial planes: sagittal, transverse and vertical. It is possible to identify three different types of skeletal relationship in the sagittal plane, defined from the analysis of the ANB angle, which represents the antero-posterior intermaxillary relationship.

Skeletal class I occurs when the ANB angle is between 0° and 4°. In this case there is a correct relationship between the upper and lower jaw, due to a harmonious growth between the jaw bases. In case of skeletal class II the ANB angle is increased over 4°; there is then an alteration of the relationship between the two maxillary bases with protruded position of the upper jaw, in relation to the mandible, a mandibular retrusion or a combination of both situations [7]. 

Finally, skeletal class III occurs when the ANB angle is less than 0°. There is an alteration in the relationship between the two maxillary bases with the mandible protruding from the upper jaw, a retrusion of the upper jaw or a combination of both conditions [8,9].

In the same plane the overjet, that is the degree of horizontal overlap of the incisal margins, can be assessed. The values vary between 2 +/– 2 mm from the occlusal plane; in case of a negative overjet the patient presents an inverted bite [10,11].

Considering the transverse plane, malocclusions that can be found are represented by contraction (crossbite) or over-expansion (scissorbite) from the occlusal plane [12].

On the vertical plane overbite represents the degree of vertical overlap of the incisal edges. The normal value is 2 mm but can change by +/– 2 mm from the vertical plane. When there are alterations in the measurement of overbite there are two types of malocclusion: open-bite, whose value is less than 0 mm; deep-bite, whose values are more than 4 mm [8,13].

Malocclusion has a multifactorial aetiology, being caused by hereditary factors, environmental factors or a combination of both [14]. Genetically determined factors exert their influence during growth and can, therefore, lead to development of a malocclusion [15]. These influences can be combined with aetiological factors such as bad habits. In cases of sucking habits the child interposes his finger, usually the thumb, between the dental arches causing the tongue to move downwards. The tongue is unable to reach its correct position on the palate, preventing it from developing transversely; moreover, the position of the thumb against the front teeth leads to their prominence. Children tend to develop an anterior open bite and posterior cross-bite due to lack of palatal development [16]. Posterior teeth may also extrude, caused by the lack of occlusal contact due to the interposition of the finger [14]. Lip or cheek sucking also causes problems with occlusion and correct skeletal-facial development. In patients with sucking of the lower lip there is contraction of the lower orbicularis and mental muscle with subsequent proinclination of the maxillary teeth, retroinclination of the mandibular teeth, increased overjet, irregularity of the lower incisors [17]. Considering oral respiration, it is generally due to nasal airway obstruction, caused, for example, by adenoid or palatine tonsil hypertrophy, rhinitis, turbinates hypertrophy. Patients affected by this condition are unable to breathe through the nose, so they breathe through the mouth with consequent dental and skeletal problems, such as open bite, clockwise rotation of the mandible, prognathism and narrow palate [18].

The persistence of malocclusion without any treatment can lead to negative problems in the quality of children’s life and their parents because of physiological and social changes, caused by this disorder [19]. There may be problems with aesthetics, mastication and phonation; according to Siluvai et al. 46% of young people with malocclusion had a negative impact on lifestyle (OHRQoL) [20].

Given the importance of malocclusion in dentistry, the aim of our study is to assess the frequency of this condition within different geographical areas.

## 2. Materials and Methods

A literature research was performed through Pubmed, Medline, Scopus, Web of Science, LILACS, Open Grey and Cochrane Library databases for this review; the keywords used for the research were “Prevalence” and “Malocclusion”.

The following inclusion and exclusion criteria were considered for studies selection:

- Studies whose patients presented an age range of 7 to 20 years.
- Studies whose patients presented mixed or permanent dentition.
- Recent studies performed from 2005 to the present.
- Studies that analysed at least two spatial dimensions.
- Exclusion of studies performed in the same geographical areas with overlapping results.
- Exclusion of systematic reviews and meta-analyses.
- Exclusion of studies whose titles correlated the prevalence of malocclusion with other problems such as temporomandibular disorders, sleep apnoea and bruxism.

The PRISMA (Preferred Reporting Items for Systematic reviews and Meta-Analyzes) guidelines were used for the following review. 

**Figure 1:**
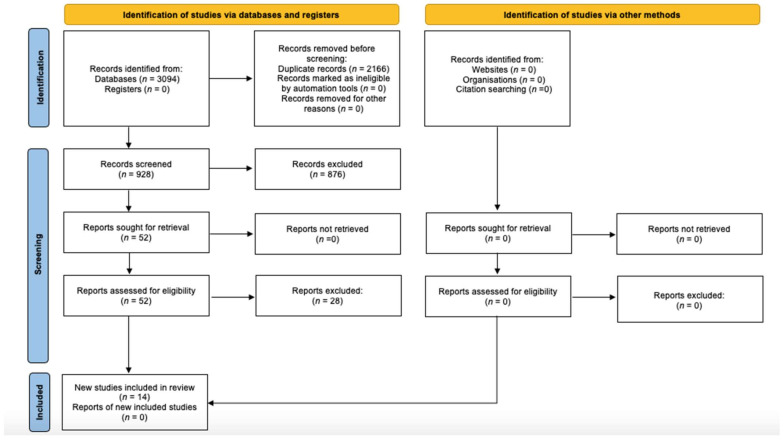
Literature search flowchart (PRISMA 2020).

A total of 1765 articles on the topic were identified within the Pubmed database and 1329 within the other search databases. All articles found were entered into EndnoteWeb^®^ to eliminate duplicates. The first phase consisted in reading the titles and eliminating all those that were not strictly relevant: many studies correlated the prevalence of malocclusion with other pathologies such as sleep apnoea or bruxism and were therefore excluded. For titles that were not clear enough to determine with certainty whether to include or exclude the article, we decided to read the abstract. This operation led to selection of 52 studies, whose materials and methods were screened. 38 of these were excluded because they did not meet the previously established selection criteria. Finally, 14 articles were included for qualitative synthesis (Figure 1).

Two reviewers independently took care of data collection and data confirmation was undertaken by a third reviewer; using an Excel spreadsheet, all the parameters analysed, useful for the aforementioned review, were entered. 

In particular, the items selected in the calculation table were divided as follows: author, title, year of publication, journal, characteristics of the sample population, class I, class II, class III, overjet, openbite, deepbite, crowding, spacing and crossbite.

### 2.1. Study Characteristics

Fourteen studies were analysed for this review. Two of them were carried out in Italy, while the remaining 10 in different countries, in order to observe possible differences in the prevalence of malocclusion in each population. Overall, the studies we selected included subjects distributed in an age range of 7 to 20 years. 

### 2.2. Study Results

The prevalence of class I occlusion according to angle ranged from 34.9% to 93.6%, class II from 4.4% to 44.7% and class III from 1.4% to 19.4%. Three studies examined the prevalence of divisions I and II within class II: division I was found in 14.5% (Mohammed et al.), 3.9% (Aikins et al.) and 40% (Gelgör et al.) of the subjects, division II in 10.7% (Mohammed et al.), 4.7% (Gelgör et al.) and 2.4% (Aikins et al.). In the third study the prevalence of division I and II was considered together and was found in 19% of patients. The frequency of increased overjet ranged from 11.8% to 63%. Reduced overjet ranged from a low of 0.4% to a high of 18.07% of the populations studied. Patients had openbite in a range of 1.4% to 35.97% and deepbite in a range of 2% to 26.3%. Subjects with crossbite ranged from 6.8% to 38.4%. Crowding ranged from a minimum of 14.4% to a maximum of 84.4%. Finally the prevalence of spacing ranged from 15.3% to 60%. 

The following Table 1, Table 2 and Table 3 show the results of the studies for each anomaly we considered.

## 3. Results

The sample of subjects found in analysed studies included scientific papers with a minimum of 155 and a maximum of 2400 subjects. 

### 3.1. Angle Classes

#### 3.1.1. Class I

Among the three classes of angle, class I was found most frequently, in a range between 34.9% (I. Gelgör) [5] and 93.6% (M. Mtaya) [2]. From the evaluation of the two studies carried out on the Italian population it is possible to observe how rather different results emerged: R. Ferro found a prevalence of 86.3% [23], whereas S. Perrotta found a prevalence of 46.8% [27]. Both studies excluded subjects with current or previously performed orthodontic treatment. However, the age range taken into consideration was different: in the first study 444 subjects over 14 years of age were considered, in the second 700 aged between 9 and 11 years. There were also differences in the geographical area, in which the work was carried out: in the first study the subjects were resident in north-eastern Italy, in the second in central Italy. As the patients studied were resident in different geographical areas, it is possible that they were subjected to different selective pressures with consequent differences in the manifestation of dental class I.

#### 3.1.2. Class II 

The average prevalence of Class II was 20.2%. This datum is in line with what was reported by G. Lombardo et al. in their review of the global assessment of malocclusion [33]. This study found a prevalence of 19.56%. However, it is interesting to note that there is a wide range in the frequency of malocclusion, varying from 4.4% (M. Mtaya) [2] to 44.7% (I. Gelgör) [5]. The analysis of the two studies conducted in India showed a different prevalence of the second class: H. Kaur et al. reported a frequency of 8.37%, whereas S. Sundareswaran reported a frequency of 17.6%. Both scientific papers are structured in the same way: they are cross-sectional studies that assessed the population living in both urban and rural areas. The age range of the patients examined is also almost the same: 13–15 years for Sundareswaran’s study and 13–17 years for H. Kaur’s study. The different prevalence of class II between the two studies can be explained by the fact that they were conducted in different states of India, the first in Karnataka, the second one in Kerala. Consequently, it is possible that the population in the two geographical areas is subject to different selective pressures and that there is ethnic heterogeneity that was not considered by the studies but could play a role in the manifestation of the problem [24,32]. Considering the subdivisions of class II E. Aikins reported a frequency of 3.9% for the first division and 2.4% for the second [22], M. Amalky found a frequency of the division I in 14.53% of the subjects and 10.7% of division II [30]; finally, I. Gelgör’s study revealed a prevalence of the division I of 40% and of the division II of 4.7% [5]. Again, the prevalence was highly variable, suggesting a difference between different countries and geographical regions, in this case Saudi Arabia, Nigeria and Anatolia.

#### 3.1.3. Class III

Class III prevalence was the least frequent of all classes of malocclusion according to angle with an average frequency of 7.2%. The countries that reported the lowest prevalence index were Italy (3.9% R. Ferro [23] and 1.6% S. Perrotta [27]), Nigeria (1.6% E. Aikins [22]) and Jordan (1.4% E. Alhaija [25]), the latter being the state that recorded the absolute lowest prevalence value among the studies we analysed. On the other hand, the countries with the highest Class III frequency were Puerto Rico (19.4% K. Alvarado [26]), Saudi Arabia (15.4% R. Gudipaneni [31]) and Brazil (14.1% E.Traebert [28]).

### 3.2. Vertical Dimension

#### 3.2.1. Openbite

The finding of openbite was highly variable, ranging from 1.4% (R. Ferro [23]) to 35.97% (H. Kaur [32]) of the samples we considered. With the exception of the studies by H. Kaur, M. Amalky (21.1%) [30], S. Perrotta (18%) [27] and M. Mtaya (16.1%) [2] who reported high prevalences of openbite, in all other cases the frequency was rather low, ranging from 1.4% in the Italian sample (R. Ferro) [23] to a maximum of 8.2% for the population living in Anatolia (I. Gelgör) [5].

#### 3.2.2. Deepbite

Deepbite has been less investigated in the studies we have examined; in fact, only six studies have evaluated this problem. The prevalence is situated in a range of values between 2% within the Italian population (S. Perrotta) [27] and 26.3% among Arab subjects (M. Amalky) [30]. Nevertheless it is interesting to note that the prevalence of 2% was only recorded in one study; for all the other ones it ranged from 14.4% [5] to 26.3% [30]. Excluding the Italian study, the frequency is rather homogeneous among the different countries examined. It was not possible in this review to make a comparison between studies carried out within the same country, since for each country only one study among those we selected took the problem of deepbite into consideration. 

#### 3.2.3. Overjet 

The increase in overjet was observed from a minimum of 11.8% [24] of the subjects up to a maximum of 63% [26]. In this case the results between the two Italian studies were superimposable: 15.6% of the patients were reported by R. Ferro had the problem [23], while 14.1% of those were reported by S. Perrotta [27]. However, there are differences in the case of reduced overjet, since R. Ferro reported a reduction in 15.2% of subjects while S. Perrotta reported a reduction in 2.2% [23,27]. The reduction of overjet in the studies examined ranged from 0.4% [29] to 18.07% [32].

#### 3.2.4. Crowding

A high percentage of patients presented dental crowding. With the exception of the Nigerian study, in which crowding was found in only 14.4% of patients [22], a prevalence of crowding of between 30% [23] and 84.4% [29] was found in all other studies. An interesting finding from F. Bourzgui’s study was that crowding occurred anteriorly in 50% of cases and only 2% posteriorly [21]. In the Lithuanian sample, on the other hand, the frequency of crowding between the upper arch (44.1%) and the lower arch (40.3%) was compared, and the results show that they are similar [29]. 

#### 3.2.5. Spacing 

Four studies looked at spacing. The prevalence range was very variable, from a minimum of 15.3% [24] to a maximum of 60% [22]. However, in view of the small number of studies evaluating this problem, further scientific work should be sought in the future in order to obtain more predictable results.

#### 3.2.6. Crossbite

Studies have shown that the prevalence of crossbite ranges from 6.8% (E. Alhaija) [25] to 38.4% (M. Amalky) [30]. A great disparity is presented between the work of M. Amalky and the other of R. Gudipaneni, both conducted in Saudi Arabia. In the latter, the prevalence was 14.2%. However, the two studies were set up differently: in the first, the subjects were simply analysed by a single examiner, whereas the second one was a cross-sectional study, which implies a difference in the structure of the two studies, which could affect the results. In addition, the two study sites are in opposite areas of Saudi Arabia, so it is possible that there is an influence in the prevalence of malocclusion between the different geographical areas [30;31]. Five studies have differentiated the crossbite into anterior and posterior: in the work of R. Ferro the anterior crossbite was found in 4% of the subjects [23] up to 27.5% in the case of S. Sundareswaran [24]. Considering the posterior crossbite, it was also quite variable, from a minimum of 0.99% (H. Kaur) [32] to a maximum of 21.4% (M. Amalky) [30].

## 4. Discussion

An analysis of the articles in the literature showed that, of the different tooth classes described by angle, the first class is the most frequent, followed by the second and finally the third class. A further useful investigation could concern the subdivision of the second class, which was little analysed and was considered only in three studies.

Within the studies examined, a prevalence of increased overjet was emphasised. The frequency of reduced overjet was low, ranging from 0.4% to 18.07%. 

Analysis in the vertical plane revealed a rather variable frequency of openbite; however, with the exception of three studies, the frequency range included small and similar values between 1.4% and 8.2%. The evaluation of deepbite, however, revealed more uniform values: excluding the study performed in Italy by S. Perrotta in which the prevalence was 2%, all the others were within a much narrower range of values. Deepbite is the anomaly in which more or less similar values were found in the various populations examined. 

The cross-sectional analysis revealed a variability in the prevalence of crossbite. Only five studies differentiated between anterior and posterior crossbite, so further studies are required to assess the different prevalences. 

Looking at the other anomalies, crowding was certainly one of the most frequent, with a prevalence of up to 84%. 

The frequency of spacing, on the other hand, was hardly analysed, with only four studies being taken into account. However, this dental problem also occurred quite frequently, reaching a prevalence of 60% in E. Aikins’ study. 

For most of the types of malocclusion we considered, the prevalence varied widely, considering different countries, and sometimes even within the same country but in distinct and distant geographical areas. This suggests a role for genetics and environmental influences, typical of each population in determining dental problems. Furthermore, none of the studies took into account the different ethnic groups that may reside within the same state and that have a different influence in determining malocclusion. Knowing the prevalence of malocclusion within a state is important and useful for predicting the likelihood of the problem occurrence. 

For this reason the collection of more prevalence data for the poorly analysed malocclusion types can be very useful in order to obtain an accurate assessment of the aforementioned conditions.

## Figures and Tables

**Table 1:** Authors, title, periodical, year and population of the articles considered.

Authors	Title	Periodical and Year	Population
1. Farid Bourzgui, Mourad Sebbar, Mouna Hamza, Laila Lazrak, Zouhair Abidine, Farid El Quars [21]	Prevalence of malocclusions and orthodontic treatment need in 8- to 12-year-old schoolchildren in Casablanca, Morocco	Progress in Orthodontics 2012	1000 subjects between 8 and 12 years old
2. E A Aikins, C O Onyeaso [22]	Prevalence of malocclusion and occlusal traits among adolescents and young adults in Rivers State, Nigeria	Odontostomatol Trop 2014	620 subjects between 13 and 20 years old
3. R Ferro, A Besostri, A Olivieri, E Stellini [23]	Prevalence of occlusal traits and orthodontic treatment needed in 14 year-old adolescents in Northeast Italy	European Journal Pediatric Dentistry 2016	444 subjects of 14 years old
4. Shobha Sundareswaran, Praveen Kizhakool [24]	Prevalence and gender distribution of malocclusion among 13–15-year-old adolescents of Kerala, South India	Indian Journal of Dental Research 2019	1554 subjects between 13 and 15 years old
5. Elham S J Abu Alhaija, Susan N Al-Khateeb, Kazem S Al-Nimri [25]	Prevalence of malocclusion in 13–15 year-old North Jordanian school children	Community Dental Health 2005	1003 subjects between 13 and 15 years old
6. Karla Alvarado, Lydia López, Rosana Hanke, Francis Picón, Sona Rivas-Tumanyan [26]	Prevalence of Malocclusion and Distribution of Occlusal Characteristics in 13- to 18-year-old Adolescents Attending Selected High Schools in the Municipality of San Juan, PR	Puerto Rico Health Science Journal 2017	155 subjects between 13 and 18 years old
7. Stefania Perrotta, Rosaria Bucci, Vittorio Simeon, Stefano Martina, Ambra Michelotti, Rosa Valletta [27]	Prevalence of malocclusion, oral parafunctions and temporomandibular disorder pain in Italian school children: an epidemiological study.	Journal of Oral Rehabilitation 2019	700 subjects between 9 and 11 years old
8. Eliane Traebert, Luiz Gustavo Teixeira Martins, Keila Cristina Raush Pereira, Simone Xavier Silva Costa, Sandra Espíndola Lunardelli, Abelardo Nunes Lunardelli, Jefferson Traebert [28]	Malocclusion in Brazilian Schoolchildren: High Prevalence and Low Impact	Oral Health and Preventive Dentistry 2015	389 subjects between 10 and 15 years old
9. Antanas Šidlauskas, Kristina Lopatiené [29]	The prevalence of malocclusion among 7–15-year-old Lithuanian schoolchildren	Medicina (Kaunas) 2009	1681 subjects between 7 and 15 years old
10. Mohammed Almalky N, Mohammad Elattar H [30]	Prevalence Of Different Types of Malocclusion Among School Children In Makkah Governorate of Saudi Arabia	International Journal of Dentistry and Oral Science 2018	289 subjects between 14 and 17 years old
11. Ravi Kumar Gudipaneni, Raed F. Aldahmeshi, Santosh R. Patil and Mohammad Khursheed Alam [31]	The prevalence of malocclusion and the need for orthodontic treatment among adolescents in the northern border region of Saudi Arabia: an epidemiological study	BMC Oral Health 2018	500 subjects between 14 and 18 years old
12. Matilda Mtaya, Anne Nordrehaug Astrøm, Pongsri Brudvik [2]	Prevalence of malocclusion and its relationship with socio-demographic factors, dental caries, and oral hygiene in 12- to 14-year-old Tanzanian schoolchildren	The European Journal of Orthodontics 2009	1601 subjects between 12 and 14 years old
13. H. Kaur, U.S. Pavithra, R. Abraham [32]	Prevalence of malocclusion among adolescents in South Indian population	Journal of International Societ of Preventive and Community Dentistry 2013	2400 subjects between 13 and 17 years old
14. Ibrahim Erhan Gelgör, Ali Ihya Karaman, Ertugrul Ercan [5]	Prevalence of malocclusion among adolescents in Central Anatolia	European Journal of Dentistry 2017	2329 subjects between 12 and 17 years old

**Table 2:** Class I, Class II, Class III, overjet, openbite prevalence for each study. (* the anomaly considered was not investigated).

	I Class	II Class	III Class	Overjet	Openbite
1	61.4%	24%	10%	63.8% 1–4 mm 17.2% 4–6 mm 10% > 6 mm	*
2	80.3%	6.3% (3.9% I° division, 2.4% II° division)	1.6%	30% (increased 15.6%; reduced 15.2%)	7.1%
3	86.3%	19.50%	3.9%	increased 48%	1.4%
4	64.3%	17.6%	8%	increased 11.8%	1.6%
5	55.3%	18.8%	1.4%	increased 24.7%, reduced nell’1.8%	2.9%
6	73%	7.10%	19.4%	increased 63%	1.9%
7	46.8%	32.6% (19% divisions)	1.6%	increased 14.1%, reduced 2.2%	18%
8	42.7%	15.9%	14.1%	30.90%	2.6%
9	68.4%	27.7%	2.8%	increased 21.09%, reduced 0.4%	3.46%
10	67.13%	I division 14.53%, II division 10.7%	7.61%	increased 25.6%, reduced 17%	21.1%
11	52.8%	31.8%	15.4%	increased 22.2% reduced 11.4%	4.6%
12	93.6%	4.4%	2%	*	16.1%
13	89.45%	8.37%	2.14%	Increased 33.71% reduced 18.07%	35.97%
14	34.9%	I division 40% II division 4.7%	10.3%	increased 25.1% reduced 10.4%	8.2%

**Table 3:** Deepbite, crowding, spacing, crossbite prevalence for each study. (* the anomaly considered was not investigated).

	Deepbite	Crowding	Spacing	Crossbite
1	*	anterior 50%, posterior 2.5%	*	*
2	*	14.4%	60%	17.1%
3	*	30%	*	anterior 4%, posterior 5%
4	*	66.6%	15.3%	anterior 27.5% posterior 5.1%
5	16.9%	50.4%	26.7%	6.80%
6	*	*	*	*
7	2%	*	*	12%
8	*	*	*	*
9	14.46%	upper 44.1%, lower 40.3%	*	8.8%
10	26.3%	63.3%	*	anterior 17%, posterior 21.4%
11	*	*	*	anterior 4.8% posterior 9.4%
12	*	*	21.9%	*
13	14.15%	58.12%	*	anterior 8.48% posterior 0.99%
14	14.4%	*	*	9.5%

## Data Availability

Not applicable.
